# What is the outcome of re-recurrent vs recurrent inguinal hernia repairs? An analysis of 16,206 patients from the Herniamed Registry

**DOI:** 10.1007/s10029-020-02138-1

**Published:** 2020-02-21

**Authors:** F. Köckerling, C. Krüger, I. Gagarkin, A. Kuthe, D. Adolf, B. Stechemesser, H. Niebuhr, D. Jacob, H. Riediger

**Affiliations:** 1 Department of Surgery and Center for Minimally Invasive Surgery, Academic Teaching Hospital of Charité Medical School, Vivantes Hospital, Neue Bergstrasse 6, 13585 Berlin, Germany; 2 Immanuel Hospital Rüdersdorf, Seebad 82/83, 15562 Rüdersdorf, Germany; 3 Spital Riggisberg, Inselgruppe,, Eyweg 2, 3132 Riggisberg, Switzerland; 4 DRK-Krankenhaus Clementinenhaus, Lützerodestr. 1, 30161 Hannover, Germany; 5 StatConsult GmbH, Halberstädter Strasse 40 a, 39112 Magdeburg, Germany; 6 Pan Hospital, Hernia Center, Zeppelinstraße 1, 50667 Köln, Germany; 7 Hansechirurgie, Niebuhr, Marleschki & Partner, Alte Holstenstr. 16, 21031 Hamburg, Germany; 8 COPV—Hernia Center, Kaiser-Wilhelm-Str. 24-26, 12247 Berlin, Germany; 9 Vivantes Humboldt Hospital, Am Nordgraben 2, 13509 Berlin, Germany

**Keywords:** Inguinal hernia, Recurrence, Re-recurrence, Outcome, Postoperative complications, Chronic pain

## Abstract

**Introduction:**

The proportion of recurrent repairs in the total collective of inguinal hernia repairs among men is 11.3–14.3% and among women 7.0–7.4%. The rate of re-recurrences is reported to be 2.9–9.2%. To date, no case series has been published on second and ≥ third recurrences and their treatment outcomes. Only case reports are available.

**Materials and methods:**

In an analysis of data from the Herniamed Registry the perioperative and 1-year follow-up outcomes of 16,206 distinct patients who had undergone first recurrent (*n* = 14,172; 87.4%), second recurrent (*n* = 1,583; 9.8%) or ≥ third recurrent (*n* = 451; 2.8%) inguinal hernia repair between September 1, 2009 and July 1, 2017 were compared.

**Results:**

The intraoperative complication rate for all recurrent repairs was between 1–2%. In the postoperative complications a continuous increase was observed (first recurrence: 3.97% vs second recurrence: 5.75% vs ≥ third recurrence 8.65%; *p* < 0.001). That applied equally to the complication-related reoperation rates (first recurrence: 1.50% vs second recurrence: 2.21% vs ≥ third recurrence 2.66; *p* = 0.020). Likewise, the re-recurrence rate rose significantly (first recurrence: 1.95% vs second recurrence: 2.72% vs ≥ third recurrence 3.77; *p* = 0.005). Similarly, the rate of pain requiring treatment rose highly significantly with an increasing number of recurrences (first recurrence: 5.21% vs second recurrence: 6.70% vs ≥ third recurrence 10.86; *p* = < 0.001).

**Conclusion:**

The repair of re-recurrences in inguinal hernia is associated with increasingly more unfavorable outcomes. For the first recurrence the guidelines should definitely be noted. For a second and ≥ third recurrence diagnostic laparoscopy may help to select the best possible surgical technique.

## Introduction

According to the guidelines of the HerniaSurge Group recurrence rates of inguinal hernia repair worldwide are still too high despite numerous innovations [1]. Recurrence rates vary in accordance with the length of follow-up [1, 2]. Recurrences after inguinal hernia repairs can occur even up to 50 years later [2]. In recent administrative data and registry analyses it was revealed that the proportion of recurrent repairs in the total collective of inguinal hernia repairs among men was 11.3–14.3% [3–7] and in women 7.0–7.4% [4, 8]. By contrast, in systematic reviews and meta-analyses the recurrence rates were still far lower (1.2–3%) than those cited above since the included studies had a maximum follow-up time of 6 years [9–11].

The guidelines of the HerniaSurge Group recommend that the first recurrence repair should be performed in an unoperated anatomic layer [1], i.e. laparo-endoscopic (TEP, TAPP) following previous open anterior repair and anterior open (Lichtenstein) following previous laparo-endoscopic repair. However, to date that recommendation is not adequately applied [5] and results in significantly higher rates of second recurrences [5].

The rates of second recurrences after recurrent inguinal hernia repair are reported in registry data and case series to be as high as 8.8% [12, 13]. In meta-analyses comparing open with laparo-endoscopic repair of first inguinal hernia recurrences the rates of second recurrences were between 2.9% and 9.2% [14–17], depending on the follow-up time.

So far, no case series has been published on second recurrences and their treatment outcomes. Only case reports are available.

Based on the analysis of data from the Herniamed Registry, this paper now compares the treatment outcomes for second recurrences and ≥ third recurrences with those of first recurrences.

## Materials and methods

The Herniamed quality assurance study is a multicenter, internet-based hernia registry [18, 19] into which 683 participating hospitals and surgeons engaged in private practice (Herniamed Study Group) in Germany, Austria and Switzerland (Status: August 1, 2018) have entered data prospectively on their patients who had undergone routine hernia surgery [20, 21]. All patients signed an informed consent agreeing to participate [20, 21]. As part of the information provided to patients regarding participation in the Herniamed Quality Assurance Study and signing the informed consent declaration, all patients are informed that the treating hospital or medical practice would like to be informed about any problem occurring after the operation and that the patient has the opportunity to attend clinical examinations [20, 21]. All postoperative complications occurring up to 30 days after surgery are recorded [20, 21]. At 1-year follow-up, postoperative complications are once again reviewed when the general practitioner and patient complete a questionnaire [20, 21]. At 1-year follow-up, the general practitioner and patient are also asked about any recurrences, pain at rest, pain on exertion, and chronic pain requiring treatment [20, 21]. If a recurrence or chronic pain is reported by the general practitioner or patient, the patient can be requested to attend clinical examination [20, 21]. One publication has provided impressive evidence of the role of patient-reported outcome for recurrence and chronic pain [22].

In the current analysis, prospective data on patients with a first recurrent, second recurrent and ≥ third recurrent elective unilateral inguinal hernia were analyzed to compare the perioperative and 1-year follow-up outcomes.

**Fig. 1:**
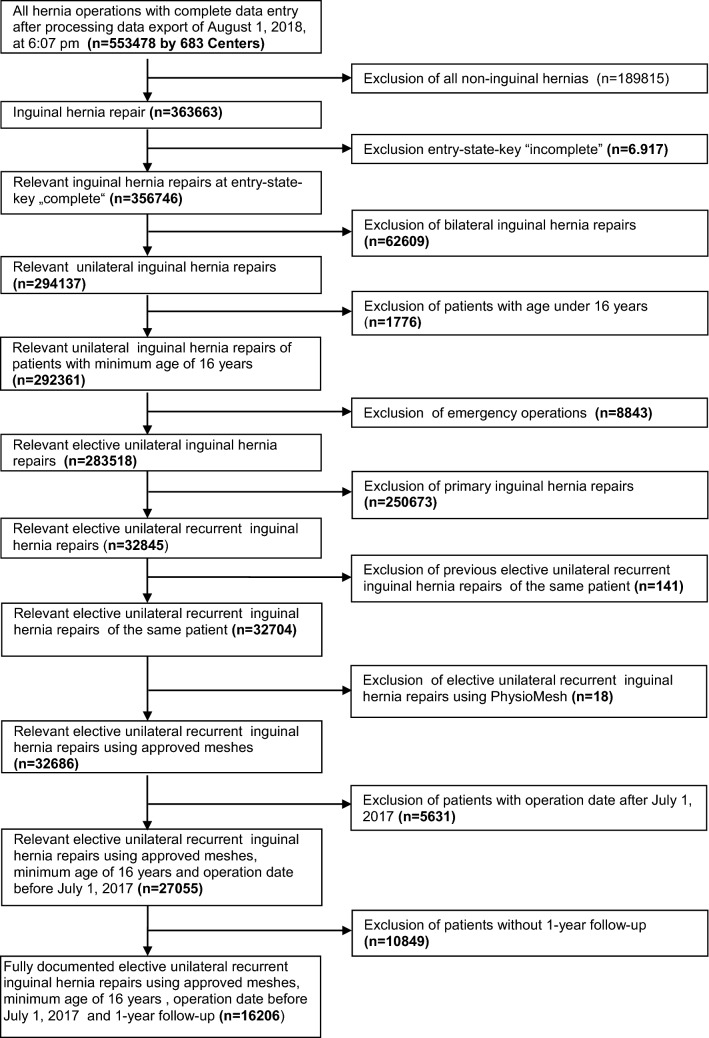
Flowchart of patients inclusion

The main inclusion criteria were minimum age of 16 years, unilateral first recurrent, second recurrent and ≥ third recurrent elective inguinal hernia repair using only the last recurrence per patient, all types of procedures, and availability of data at 1-year follow-up (Fig. 1).

All analyses were performed with the software SAS 9.4 (SAS Institute Inc., Cary, NC, USA) and intentionally calculated to a full significance level of 5%, i.e. no corrections were made for multiple testing and each *p* value ≤ 0.05 corresponds to a significant result.

Individual outcome and influence variables (risk factors, complications) were summarized as global variables. A general, intra- or postoperative complication or risk factor was deemed to apply if at least one such individual item was present.

Therefore, all categorical patient data are presented as absolute and relative frequencies for these categories in contingency tables.

For continuous data the mean value and standard deviation or, for log-transformed data, the mean value and range are presented.

For analysis of an individual influence variable on an individual outcome parameter unadjusted analyses were carried out. Here the focus was on the influence exerted by the first recurrent, second recurrent and ≥ third recurrent repair.

The chi-square test was performed for categorical outcome variables. ANOVA (analysis of variance) was used for continuous variables to analyze the influence exerted by the comparison groups.

## Results

In total, 16,206 patients were selected between September 1, 2009 and July 1, 2017 (Fig. 1). Following patient selection 16,206 patients were ultimately included in the analysis comparing the outcomes for first recurrent, second recurrent and ≥ third recurrent inguinal hernia repair. Of these patients, 14,172 (87.4%) underwent first recurrent, 1,583 (9.8%) patients second recurrent and 451(2.8%) patients ≥ third recurrent inguinal hernia repair (Table 1).

**Table 1:** Patient population with 1. recurrence, 2. recurrence and ≥ 3. recurrence

	*N*	%
1. Recurrence	14,172	87.4
2. Recurrence	1583	9.8
≥ 3. Recurrence	451	2.8
Total	16,206	100.0

Table 2 presents the descriptive statistics as well as the test results for the continuous variables age, BMI and operating time. While there are significant differences because of the large sample size, only the difference in the operating time is clinically relevant.

**Table 2:** Comparison of mean age, BMI and operation-time in patients with 1. recurrent versus 2. recurrent versus ≥ 3. recurrent inguinal hernia repair

		1. Recurrence	2. Recurrence	≥ 3. Recurrence	*p*
Age (years)	*N*/mean ± STD	14,172/61.2 ± 15.2	1583/62.8 ± 14.5	451 / 62.1 ± 14.3	< .001
BMI (kg/m^2^)	*N*/mean ± STD	14,121/25.9 ± 3.5	1573/26.0 ± 3.6	450 / 26.4 ± 3.8	0.008
Log Operation-time [min]	*N*/MW[range]	14,029/52.3 [50.8; 53.8]	1573/57.3 [55.8; 58.9]	447 / 61.0 [59.3; 62.6]	< .001

Unadjusted analysis of the relationship between the first recurrent, second recurrent and ≥ third recurrent repair and the patient- and surgery-related variables (Table 3) revealed major differences, with the exception of the scrotal EHS classifications and most risk factors. The proportion of women rose significantly in line with the increase in the number of recurrences.

**Table 3:** Comparison of demographic and surgery-related parameters and risk factors of patients with 1. recurrent versus 2. recurrent versus ≥ 3. recurrent inguinal hernia repair

	1. Recurrence		2. Recurrence		≥ 3. Recurrence		*p*
	*n*	%	*n*	%	*n*	%	
Gender							
Male	13,010	91.80	1437	90.78	399	88.47	0.019
Female	1162	8.20	146	9.22	52	11.53	
Procedure							
Bassini	37	0.26	11	0.69	3	0.67	< .001
Defect closure	4	0.03	0	0.00	1	0.22	
Gilbert	160	1.13	21	1.33	1	0.22	
Lichtenstein	5283	37.28	581	36.70	167	37.03	
Plug	525	3.70	68	4.30	17	3.77	
Shouldice	259	1.83	30	1.90	10	2.22	
Sonstige	281	1.98	63	3.98	23	5.10	
TAPP	5012	35.37	555	35.06	153	33.92	
TEP	2416	17.05	222	14.02	60	13.30	
TIPP	195	1.38	32	2.02	16	3.55	
ASA score							
I	3723	26.27	343	21.67	92	20.40	< .001
II	7893	55.69	934	59.00	263	58.31	
III/IV	2556	18.04	306	19.33	96	21.29	
Defect size							
I (< 1.5 cm)	2897	20.44	316	19.96	99	21.95	0.020
II (1.5—3 cm)	8136	57.41	868	54.83	237	52.55	
III (> 3 cm)	3139	22.15	399	25.21	115	25.50	
EHS-classification medial							
Yes	7312	51.59	899	56.79	254	56.32	< .001
No	6860	48.41	684	43.21	197	43.68	
EHS-classification lateral							
Yes	8208	57.92	796	50.28	226	50.11	< .001
No	5964	42.08	787	49.72	225	49.89	
EHS-classification femoral							
Yes	504	3.56	98	6.19	28	6.21	< .001
No	13,668	96.44	1485	93.81	423	93.79	
EHS-classification scrotal							
Yes	280	1.98	26	1.64	13	2.88	0.243
No	13,892	98.02	1557	98.36	438	97.12	
Preoperative pain							
Yes	9160	64.63	1093	69.05	325	72.06	< .001
No	3797	26.79	371	23.44	78	17.29	
Unknown	1215	8.57	119	7.52	48	10.64	
Drainage							
Yes	4305	30.38	588	37.14	198	43.90	< .001
No	9867	69.62	995	62.86	253	56.10	
Risk factors							
Yes	4440	31.33	547	34.55	164	36.36	0.003
No	9732	68.67	1036	65.45	287	63.64	
COPD							
Yes	864	6.10	132	8.34	31	6.87	0.002
No	13,308	93.90	1451	91.66	420	93.13	
Diabetes							
Yes	848	5.98	100	6.32	30	6.65	0.744
No	13,324	94.02	1483	93.68	421	93.35	
Aortic aneurysm							
Yes	100	0.71	7	0.44	4	0.89	0.421
No	14,072	99.29	1576	99.56	447	99.11	
Immunosuppression							
Yes	132	0.93	9	0.57	3	0.67	0.302
No	14,040	99.07	1574	99.43	448	99.33	
Corticoid treatment							
Yes	164	1.16	22	1.39	3	0.67	0.432
No	14,008	98.84	1561	98.61	448	99.33	
Smoking							
Yes	1552	10.95	203	12.82	60	13.30	0.029
No	12,620	89.05	1380	87.18	391	86.70	
Coagulopathy							
Yes	270	1.91	24	1.52	12	2.66	0.265
No	13,902	98.09	1559	98.48	439	97.34	
ASS/Plavix Antiplatelet medication							
Yes	1448	10.22	155	9.79	51	11.31	0.639
No	12,724	89.78	1428	90.21	400	88.69	
Anticoagulation therapy							
Yes	393	2.77	46	2.91	19	4.21	0.188
No	13,779	97.23	1537	97.09	432	95.79	

As regards the surgical techniques, the standard procedures TEP, TAPP and Lichtenstein declined somewhat in line with the rising number of recurrences, while the TIPP and other procedures increased.

Patients with a second recurrence or ≥ third recurrence had a significantly higher ASA score and EHS III defect size (> 3 cm).

The proportion of medial and femoral EHS classifications rose significantly for second recurrences and ≥ third recurrences.

Preoperative pain was identified significantly more often for second recurrences and ≥ third recurrences.

The rate of risk factors (COPD, diabetes, aortic aneurysm, immunosuppression, corticoidsteroid therapy, smoking, coagulopathy, antiplateled medication and anticoagulation therapy) was significantly increased for second recurrences and ≥ third recurrences compared with first recurrences (Table 3).

Significant differences were identified for all outcome variables in relation to the number of instances of recurrence with the exception of intraoperative complications (Table 4).

**Table 4:** Comparison of perioperative and 1-year follow-up outcomes in patients with 1. recurrent versus 2. recurrent versus ≥ 3. recurrent inguinal hernia repair

	1. Recurrence		2. Recurrence		≥ 3. Recurrence		
	*n*	%	*n*	%	*n*	*%*	*p*
Intraoperative complication							
Yes	187	1.32	29	1.83	7	1.55	0.239
No	13,985	98.68	1554	98.17	444	98.45	
Postoperative complication							
Yes	563	3.97	91	5.75	39	8.65	<.001
No	13,609	96.03	1492	94.25	412	91.35	
Complication-related reoperation							
Yes	213	1.50	35	2.21	12	2.66	0.020
No	13,959	98.50	1548	97.79	439	97.34	
Recurrence on 1-year-follow-up							
Yes	277	1.95	43	2.72	17	3.77	0.005
No	13,895	98.05	1540	97.28	434	96.23	
Pain on exertion on 1-year-follow-up							
Yes	2110	14.89	326	20.59	107	23.73	<.001
No	12,062	85.11	1257	79.41	344	76.27	
Pain in rest on 1-year-follow-up							
Yes	1170	8.26	156	9.85	53	11.75	0.004
No	13,002	91.74	1427	90.15	398	88.25	
Pain requiring treatment on 1-year-follow-up							
Yes	738	5.21	106	6.70	49	10.86	< .001
No	13,434	94.79	1477	93.30	402	89.14	

Accordingly, the postoperative complication rate rose significantly from 3.97% for the first recurrence to 5.75% for the second recurrence and to 8.65% for the ≥ third recurrence (*p* < 0.001). That was also true for the complication-related reoperation rate (1.50% vs 2.21% vs 2.66%; *p* = 0.020). Likewise, re-recurrence increased significantly in relation to previous recurrences at 1-year follow-up (1.95% vs 2.72% vs 3.77%; *p* = 0.005). Pain at rest (*p* = 0.004), pain on exertion (*p* < 0.001) and chronic pain requiring treatment (*p* < 0.001) also rose significantly with each additional recurrence (Table 4). Chronic pain requiring treatment for the first recurrence was 5.21%, for the second recurrence 6.70% and for the ≥ third recurrence 10.86%.

Additional subgroup analysis of patients missing to follow-up.

**Fig. 2:**
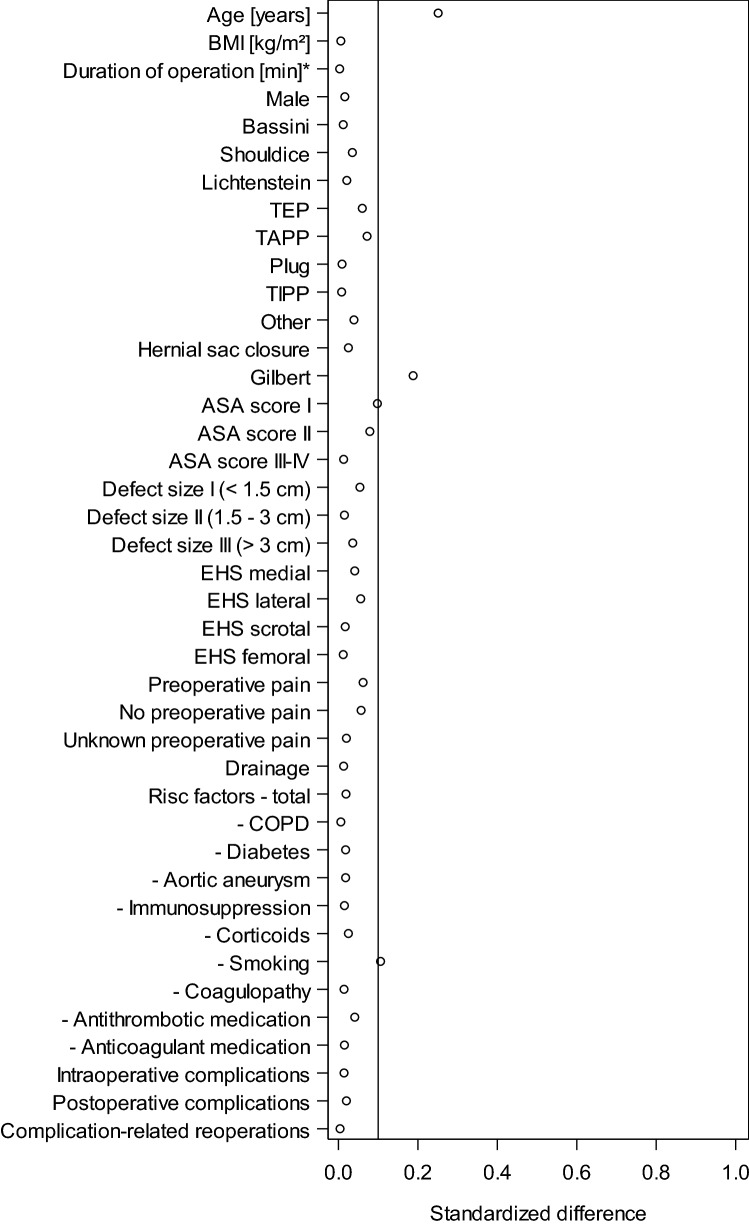
Standardized differences of the influencing factors and the perioperative outcomes between patient collectives with and without follow-up

To rule out selection bias patient subgroups with and without follow-up were compared with regard to influence factors and perioperative outcomes. The standardized differences showed a difference of > 0.1 only for the mean age and the proportion of Gilbert repairs (Fig. 2). Since no relevant deviations were noted for any of the other influence factors or for the perioperative outcome, selection bias can be neglected.

## Discussion

Analysis of 16,206 inguinal hernia recurrent repairs revealed a proportion of 87.4% for first recurrences, 9.8% for second recurrences, and 2.8% for ≥ third recurrences. For the intraoperative complications the number of instances of recurrence was not found to have any significant influence on the outcome. By contrast, a significantly increasing rate of postoperative complications of up to 8.65% was identified for ≥ third recurrences. Likewise, the complication-related reoperation rate rose to 2.66% for ≥ third recurrences. The re-recurrence rate at 1-year follow-up also increased to 3.77% in patients with ≥ third recurrences. The number of instances of recurrence also had a greater influence on the pain rates. For example, for each additional recurrence the pain on exertion rate rose significantly to 23.73%, the pain at rest rate to 11.75% and the rate of chronic pain requiring treatment to 10.86%.

Due to the relatively small number of re-recurrences, data analyses were limited to tests unadjusted for potential confounders here. Thus, estimated differences in outcome between comparison groups may further be influenced by patient- and operation-related characteristics.

Nevertheless, to date, there are no comparable findings for these data in the literature. Only the proportion of re-recurrences of around 8% is also seen in the Danish Hernia Database [11]. The data impressively demonstrate just how demanding is inguinal hernia surgery for recurrences and re-recurrences. It requires extensive experience to avoid perioperative complications, re-recurrences and chronic pain rates. Therefore, according to the HerniaSurge Guidelines [1], an expert hernia surgeon should repair a recurrent inguinal hernia after a failed anterior and posterior repair. The HerniaSurge guidelines recommend for recurrence after failed posterior repair an anterior open technique (Lichtenstein) and a laparo-endoscopic repair (TEP, TAPP) after failed anterior tissue or Lichtenstein repair [1].

For second and ≥ third recurrences surgeons have used the standard procedures TEP, TAPP and Lichtenstein less, opting instead for the open preperitoneal and other techniques. The same trend was observed in the registry analysis of the Danish Hernia Database [12].

Important is the finding of more medial and femoral recurrent inguinal hernias with each succeeding repair. This may reflect a reluctance to place larger meshes with more medial overlap especialy at open repair and occult or missed primary femoral hernia present at the index or recurrent operation.

Diagnostic laparoscopy can be useful for second recurrences and ≥ third recurrences to decide which surgical access route offers the best outcome prospects for repair of a re-recurrence [23, 24]. The laparoscopy findings will make a valuable contribution when deciding whether a laparo-endoscopic procedure or an open technique assures better conditions.

In view of the very unfavorable outcomes observed for second recurrent and ≥ third recurrent inguinal hernias, that additional investment is also justified. Only such supplementary diagnostic measures are able to improve the unfavorable outcomes for repair of second recurrent and ≥ third recurrent inguinal hernias, which should always be performed as mesh supported repair. Sometimes very individual solutions are necessary to treat a re-recurrent hernia [25].

What is true for a first inguinal hernia recurrence [1] is all the more true for a second and ≥ third inguinal hernia recurrence. Such a repair should only be undertaken by a highly experienced hernia surgeon while utilizing all diagnostic aids. The surgeon should have the necessary experience of all relevant surgical techniques (TEP, TAPP, Lichtenstein, open preperitoneal mesh).

Incorrect or missing data limit a registry [20]. Hospitals and surgeons participating in the Herniamed Registry sign a contract for data correctness and completeness [20]. As part of the certification process of hernia centers, experts control data entry [20].

On comparing the patient subgroups with and without 1-year follow-up to exclude selection bias, a standardized difference of > 0.1 was found only for the mean age and the proportion of Gilbert repairs. All other potential influence factors and the perioperative outcomes were comparable.

In summary, unadjusted comparison of the perioperative and 1-year follow-up outcomes for first recurrent vs second recurrent vs ≥ third recurrent inguinal hernia repairs showed significantly unfavorable results. Therefore, the guidelines should definitely be followed for the first recurrent inguinal hernia repair in order to avoid further recurrences. Furthermore, inguinal hernia recurrences should only be repaired by highly experienced hernia surgeons. Diagnostic laparoscopy can help to select the best possible procedure for the individual patient.
